# Nutrient-rich environments drive microbiome restructuring and mucus shedding in a coastal cnidarian

**DOI:** 10.3389/fmicb.2026.1792133

**Published:** 2026-05-18

**Authors:** Tim Lachnit

**Affiliations:** Zoological Institute, Christian-Albrechts-Universität, Kiel, Germany

**Keywords:** dysbiosis, eutrophication, host defense, host–microbe interaction, imbalance microbiota, microbiome, mucus shedding

## Abstract

**Introduction:**

Anthropogenic nutrient enrichment is increasing in coastal ecosystems, yet its effects on host–microbe interactions and animal fitness remain insufficiently understood. This study investigates how elevated dissolved nutrient availability and differing nutrient compositions influence microbiome structure and host responses in the sea anemone Nematostella vectensis.

**Methods:**

We experimentally enriched seawater with either a complex organic nutrient source or a protein-rich substrate. We then assessed resulting changes in microbiome composition, host behavior (including mucus shedding), and population growth.

**Results:**

Nutrient enrichment caused substantial restructuring of the host-associated microbiome, characterized by reduced diversity and increased dominance of fast-growing bacterial taxa. Community composition differed depending on nutrient type. Enriched conditions also triggered increased ectodermal mucus shedding, which physically removed surface-associated microbes. Copiotrophic taxa that proliferated under nutrient enrichment were disproportionately represented in shed mucus relative to whole polyps, suggesting spatial structuring of microbial associations with host surfaces. While this response limited microbial overgrowth, it imposed significant physiological costs, leading to reduced or negative population growth.

**Discussion:**

These findings demonstrate that nutrient enrichment alters host-associated microbiomes and identify mucus shedding as a host-mediated mechanism for microbial regulation. Although effective, this response carries substantial fitness costs, highlighting a trade-off between microbial control and host performance. This study provides mechanistic insight into how eutrophication may drive microbiome restructuring and impact host physiology in coastal cnidarians.

## 1 Introduction

Anthropogenic nutrient enrichment is a pervasive and escalating feature of aquatic ecosystems worldwide, particularly in coastal and estuarine environments that are increasingly exposed to elevated inputs of dissolved organic matter, nitrogen, and phosphorus as a consequence of agricultural runoff, wastewater discharge, and climate-driven changes in precipitation patterns (Kennish, 2002; Bricker et al., 2008; Malone and Newton, 2020). These processes have transformed many formerly oligotrophic or mesotrophic systems into eutrophic conditions, with far-reaching consequences for ecosystem structure and function, including altered primary production and biogeochemical cycling (Kennish, 2002; Deininger and Frigstad, 2019). While the ecological impacts of eutrophication on primary producers and biogeochemical cycles are well documented, its effects on host–microbe interactions and animal health remain less well understood (Harvell et al., 1999; Lafferty et al., 2004; Ward and Lafferty, 2004; Egan and Gardiner, 2016; Diner et al., 2024).

All multicellular organisms live in intimate association with diverse microbial communities, forming functional units often referred to as holobionts (Rosenberg et al., 2007; Bosch and McFall-Ngai, 2011; Deines et al., 2017; Jaspers et al., 2019). These host-associated microbiomes contribute to nutrition, development, immunity, and protection against pathogens across a wide range of hosts and environments (Gould et al., 2018; Parfrey et al., 2018; Bernardo-Cravo et al., 2020). Across both terrestrial and aquatic systems, disruption of these communities; commonly termed dysbiosis; has been linked to disease development, reduced fitness, and increased susceptibility to environmental stress, as shown for animals, plants and humans (Connelly et al., 2022; Li et al., 2022; Botté et al., 2023; Greene et al., 2023; Winter and Bäumler, 2023). However, a central unresolved question is whether dysbiosis is a cause or consequence of disease, and which environmental drivers initiate the transition from stable host–microbe associations to maladaptive or pathogenic states, as emphasized in conceptual work on marine disease ecology and microbial dysbiosis (Lafferty et al., 2004; Egan and Gardiner, 2016).

One emerging framework to explain environmentally driven dysbiosis is the “overfeeding hypothesis,” which proposes that many host-associated microbes are evolutionarily adapted to nutrient-limited conditions and rely primarily on host-derived metabolites for growth (Lachnit et al., 2019). Under such conditions, microbial proliferation is indirectly regulated by the host through control of nutrient secretion. External nutrient enrichment can uncouple this dependency by providing readily available substrates from the environment, thereby favoring fast-growing, opportunistic microbes over host-adapted symbionts (Chakraborty, 2024). This shift can lead to uncontrolled microbial growth, altered community composition, and functional changes that destabilize host–microbe homeostasis (Côte et al., 2022; Klinges et al., 2023; Lachnit et al., 2025). This pattern aligns with widespread disease outbreaks and microbiome restructuring observed across diverse marine hosts exposed to anthropogenic nutrient enrichment, including corals, molluscs, seagrasses, and fish (Wang et al., 2018; Hudson and Egan, 2024; Pulkkinen and Taskinen, 2024; Liu et al., 2025). Although evidence from both freshwater and marine systems links coastal eutrophication to shifts in host-associated microbiota, the mechanistic pathways connecting nutrient enrichment to microbiome restructuring, host responses, and fitness consequences remain incompletely resolved.

Cnidarians provide powerful model systems to study these processes, as their microbial communities are largely associated with external epithelial surfaces and are therefore directly exposed to surrounding environmental conditions (Deines et al., 2017). The sea anemone *Nematostella vectensis* is an established model for host–microbe interaction, possessing a relatively stable core microbiome that exhibits plasticity based on host genotype and environmental parameters such as temperature and salinity (Mortzfeld et al., 2016; Baldassarre et al., 2022, 2023; Domin et al., 2023). However, despite this growing knowledge of microbiome plasticity, previous studies have not experimentally isolated dissolved nutrient enrichment as a direct driver of dysbiosis in this system. It remains unclear whether elevated nutrient availability alone is sufficient to restructure the *Nematostella* microbiome and elicit compensatory host responses. Building on the evolutionary and ecological framework that exposure of host-associated microbiomes to nutrient-rich conditions may lead to dysbiosis and disease in the freshwater polyp *Hydra* (Lachnit et al., 2025), we investigate how elevated nutrient availability and nutrient composition affect the microbiome of the sea anemone *Nematostella vectensis*. By experimentally enriching seawater with either a complex organic nutrient source or a protein-rich substrate, we test whether nutrient load and quality differentially structure bacterial communities, promote dysbiosis, and impose physiological costs on the host.

## 2 Materials and methods

### 2.1 Culture conditions and experimental setup

Asexually reproducing *Nematostella vectensis* polyps were used for all experiments. The linage originated from the F1 offspring of CH2XCH6 individuals collected from the Rhode River in Maryland, USA (Hand and Uhlinger, 1992; Fritzenwanker and Technau, 2002). Polyps were maintained at 20 °C in the dark in *Nematostella* medium (Milli-Q water supplemented with 16‰ Red Sea Salt^®^). Before experimental start polyps were fed twice weekly with an excess of first instar nauplius larvae of *Artemia salina* (∼50 per polyp; Ocean Nutrition Micro *Artemia* Cysts 430-500 gr, Coralsands, Wiesbaden, Germany). *Artemia* cysts were hatched for 24 h in 38‰ artificial seawater at 30 °C under aeration and continuous light. Nauplii were collected by filtration and rinsed twice in in *Nematostella* medium before administration. Six hours post-feeding, polyps were washed in *Nematostella* medium to remove excess prey.

### 2.2 Impact of dissolved nutrients on bacterial community composition

To test the impact of elevated nutrients on *Nematostella* host–microbe homeostasis, polyps were exposed to one of the three treatments prepared in artificial seawater (Milli-Q water supplemented with 16‰ Red Sea Salt^®^): (I) complex nutrient source (C^+^), R2A broth bacterial culture medium (ROTH^®^, 0.3 mg/ml); (II) protein source (P^+^), peptone (0.05 mg/ml) and (III) a control consisting of artificial seawater only. R2A broth and protein source were used as a proxy for complex dissolved organic nutrient enrichment. The concentrations used in this study are within the range of values reported for naturally occurring eutrophic environments. The highest reported DOC concentration, 0.36 mg/mL, was observed in a heavily impacted upper estuary receiving primary-treated pulp mill effluent. By comparison, DOC concentrations in the North Sea and Baltic Sea range from 0.004 to 0.027 mg/mL (Lønborg et al., 2024). Assuming that dissolved organic matter (DOM) contains approximately 50% carbon by mass, these DOC values correspond to estimated DOM concentrations of approximately 0.72 mg/mL and 0.008–0.054 mg/mL, respectively.

At the start of the experiment, each container was stocked with three individuals. Experiments were performed in separate containers with five biological replicates per treatment (*n* = 5) to ensure experimental independence. To maintain water quality and stable nutrient concentrations, medium and containers were replaced daily. All treatments were replicated (*n* = 5) in separate containers to ensure experimental independence. Notably, polyps were not fed during this experiment, with the last feeding occurring 72 h prior to the experimental start. At 24, 48 and 72 h, 5 individuals per treatment were collected for bacterial community analysis.

### 2.3 Population growth experiment

To assess the effects of elevated dissolved nutrients on population growth, we used the same experimental setup described above. Each replicate container was initially stocked with three *Nematostella* individuals in either C^+^, P^+^, or control medium. In contrast to the microbiome experiment, polyps in the growth trial were fed twice weekly with freshly hatched A. salina nauplii. A concentrated larval suspension (5 mL) was added to each container to ensure food surplus (∼50 nauplii per polyp), and excess larvae were removed after 6 h. To prevent biofilm accumulation and maintain environmental stability over the 27–day experimental period, the medium was continuously exchanged via a flow-through system with an hourly renewal rate, and containers were replaced daily to prevent biofilm accumulation on vessel surfaces. Population size (total number of individuals per container) was recorded every 2–3 days over a 27–day experimental period. Population size data were analyzed using a mixed-effects model (REML) with treatment and time as fixed effects and replicate container as a random effect.

### 2.4 Quantification of mucus shedding and bacterial community analysis

Under elevated nutrient conditions, we observed increased ectodermal mucus shedding, during which the external mucus layer detached from the body column and was released as a distinct ring-shaped structure at the oral end of the polyp. To quantify this behavior, we repeated the initial experiment and recorded every shedding event for individual polyps over a 72 h period. In parallel, a second experiment was performed under continuous observation to enable the separate collection of freshly shed polyps and their shed mucus layers for subsequent bacterial community analysis.

### 2.5 DNA extraction, sequencing and statistical analysis

DNA was extracted from whole polyps or shed mucus rings by using the DNA Blood and Tissue Kit (Qiagen). Prior to extraction, samples were rinsed twice in sterile filtered and autoclaved artificial seawater to remove loosely associated microbes. Microbial community composition was determined by amplicon sequencing of the variable region V1–V2 of the 16S rRNA gene. We used the following primers: Forward primer 27F: (5’-AATGATACGGCGACCACCGAGATCTACAC XXXXXXXX TATGGTAATTGT AGAGTTTGATCCTGGCTCAG-3’). Reverse primer 338R: (5’-CAAGCAGAAGACGGCATACGAGAT XXXX XXXX AGTCAGTCAGCC TGCTGCCTCCCGTAGGAGT-3’). Primers contained the Illumina adapter p5 (forward) and p7 (reverse) and unique MIDs (designated as XXXXXXXX) to label each PCR product. PCR reactions were performed in duplicate using Phusion Hot Start DNA Polymerase (Finnzymes, Espoo, Finland). PCR cycling conditions were: 98 °C for 30 s, 30 × [98 °C–9 s, 55 °C–30 s, 72 °C–90 s], 72 °C–10 min. PCR products were combined and purified by using the MinELute Gel Extraction Kit (Qiagen) after agarose gel electrophoresis. Sequencing was performed on the Illumina MiSeq platform at the sequencing facility of the Kiel Institute for Clinical Molecular Biology (IKMB). Sequencing data was analyzed using the MOTHUR packages (Schloss et al., 2009) according to the MiSeq SOP (Kozich et al., 2013). In summary, MiSeq paired-end reads were assembled and quality-controlled resulting in 4,000 sequences per sample. Sequences were grouped into operational taxonomic units (OTUs) using a 97% similarity threshold. Sequences were aligned to the SILVA 128 Database and taxonomically classified by the RDP classifier. Multidimensional scaling analysis of OTU abundance data based on Bray–Curtis similarity was performed by the Primer software Version 7.0.13 (Primer-E) (Anderson et al., 2008). PERMANOVA was used to test for significant differences between the different treatment groups. To identify the OTUs contributing most strongly to differences in community composition among treatments, SIMPER analysis based on Bray–Curtis dissimilarity was performed in Primer Version 7.0.13 (Primer-E). SIMPER was used to quantify the contribution of individual OTUs to the average dissimilarity between control and nutrient-enriched treatments, as well as between the two enrichment regimes. Raw data are deposited in the Sequence Read Archive (SRA) and are available under project ID SRX31876269-SRX31876193.

We analyzed and visualized our data using GraphPad Prism 9.3.1. Bacterial growth was plotted with error bars indicating standard error. Normality of data was determined using the Shapiro–Wilk test, and homogeneity of variances was evaluated with Bartlett’s test. Statistical significance was evaluated using one-way ANOVA followed by Šidák’s multiple comparisons test when assumptions of normality and equal variances were fulfilled. The significance threshold was set at α = 0.05. Data that did not meet these criteria were log-transformed (LN) prior to analysis. If LN-transformed data still violated normality or variance homogeneity, non-parametric Kruskal–Wallis tests followed by Dunn’s multiple comparisons test were applied.

## 3 Results

### 3.1 Nutrient enrichment alters bacterial community composition

**FIGURE 1:**
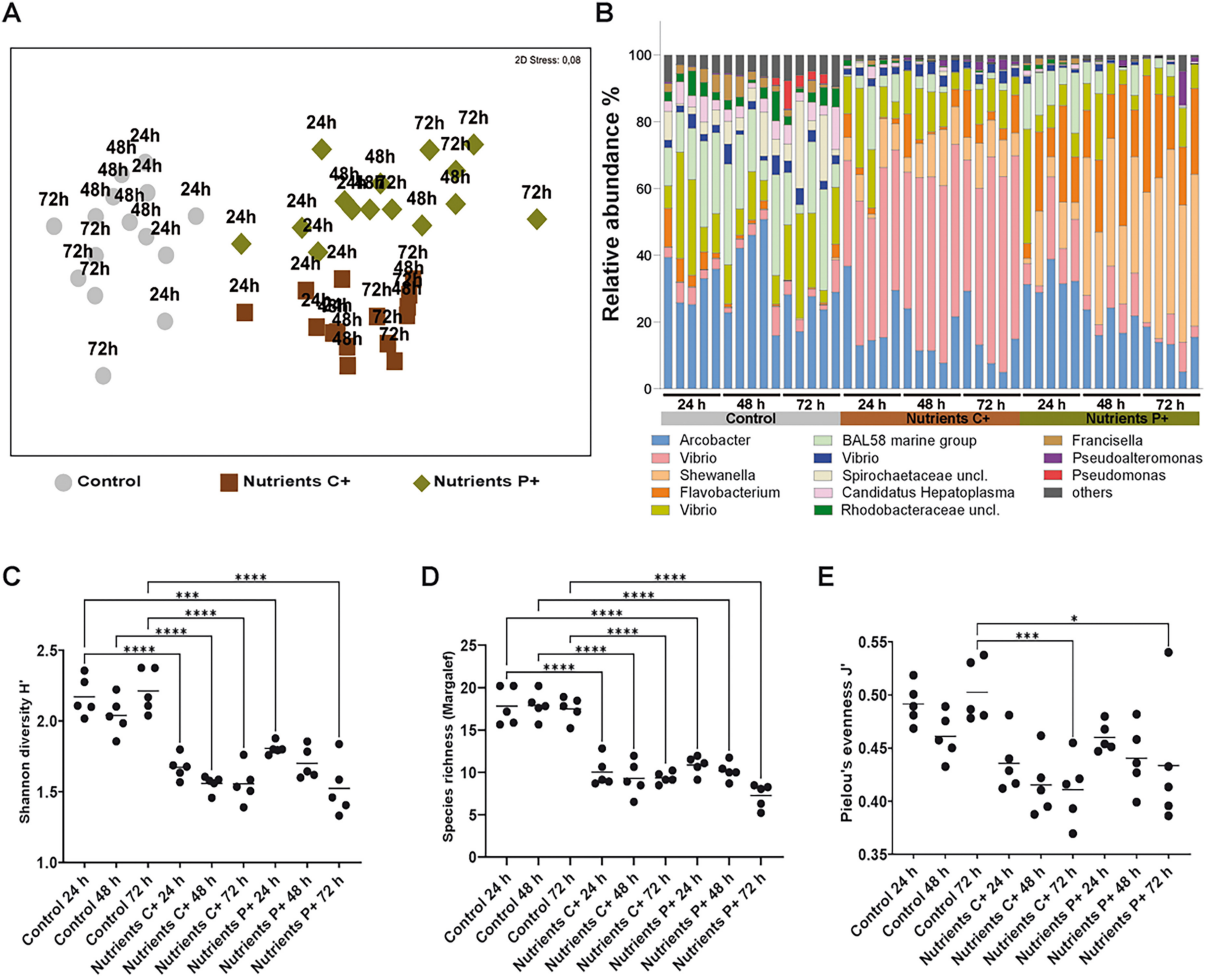
Elevated nutrient availability and composition restructure the bacterial community of *Nematostella vectensis*. **(A)** Non-metric multidimensional scaling analysis (nMDS) of *Nematostella*-associated microbial communities based on 16S rRNA gene amplicon sequencing, comparing clean seawater controls with polyps exposed to complex medium (C^+^) and protein-rich medium (P^+^). Both nutrient-enriched treatments formed distinct clusters relative to controls and separated from each other, indicating treatment-specific effects on community composition (PERMANOVA, *p* = 0.001). **(B)** Bar chart illustrating the relative abundance of dominant bacterial operational taxonomic units (OTUs) across treatments. Nutrient enrichment increased the abundance of copiotrophic taxa such as *Shewanella*, *Vibrio*, and *Flavobacterium*, while reducing the relative abundance of the R262 marine group, *Spirochaetota*, and *Candidatus Hepatoplasma*. **(C,D)** Microbial diversity declined under nutrient enrichment, as indicated by significantly lower Shannon diversity **(C)** and observed richness **(D)** in both C^+^ and P^+^ treatments compared with controls across all time points (Šidák’s test, most samples: *p* < 0.0001, except P^+^ at 24 h: *p* = 0.0009). **(E)** Pielou’s evenness was reduced under nutrient-enriched conditions, with significant differences emerging after 72 h (C^+^: *p* = 0.0004; P^+^: *p* = 0.0103). The symbols *, **, ***, and **** denote statistical significance at *p* < 0.05, *p* < 0.01, *p* < 0.001, and *p* < 0.0001, respectively.

Exposure of *Nematostella* to elevated nutrient concentrations in the surrounding seawater altered bacterial community composition. PERMANOVA detected significant differences between each enrichment treatment and the control at all time points (24 h: C^+^, *t* = 3.24, *p* = 0.005; P^+^, *t* = 2.73, *p* = 0.005; 48 h: C^+^, *t* = 5.50, *p* = 0.012; P^+^, *t* = 5.97, *p* = 0.005; 72 h: C^+^, *t* = 5.67, *p* = 0.006; P^+^, *t* = 5.85, *p* = 0.004), as well as significant differences between the C^+^ and P^+^ treatments (24 h: *t* = 1.93, *p* = 0.011; 48 h: *t* = 2.72, *p* = 0.012; 72 h: *t* = 3.12, *p* = 0.012). These differences are visualized by Multidimensional scaling (MDS) analyses (Figure 1A), which shows separation between control samples and both nutrient-enriched treatments, as well as between the two enrichment regimes.

SIMPER analysis revealed that more than 50% of the dissimilarity between nutrient-enriched treatments and controls was attributable to changes in the relative abundance of six dominant OTUs (Figure 1B and Supplementary Table 1). Three taxa increased markedly under nutrient enrichment: *Shewanella* (from 0.75% in controls to 12.6% in C^+^ and 18.02% in P^+^), *Vibrio* (from 6.08% to 21.56% in C^+^ and 10.95% in P^+^), and *Flavobacterium* (from 4.14% to 8.2% in C^+^ and 16.73% in P^+^). In contrast, three OTUs declined substantially in enriched conditions: the *BAL58 marine group* (from 11.35% to 6.06% in C^+^ and 8.23% in P^+^), *Spirochaetaceae* uncl. (from 7.34% to 1.33% in C^+^ and 0.73% in P^+^), and *Candidatus Hepatoplasma* (from 6.67% to 2.19% in C^+^ and 1.65% in P^+^).

Comparison between the two nutrient-enriched treatments indicated that approximately 50% of the dissimilarity between C^+^ and P^+^ was explained by a few taxa: *Vibrio* was relatively more abundant in C^+^, whereas *Flavobacterium*, *Shewanella*, and the *RS62 marine group* were enriched in P^+^ (Figure 1B). These shifts highlight treatment-specific effects of nutrient composition on dominant bacterial taxa.

Nutrient enrichment significantly reduced microbial diversity, as indicated by lower observed richness and Shannon diversity in both enriched treatments compared to controls at all time points (Figures 1C, D; Šidák’s multiple comparisons test, *p* < 0.0001, except P^+^ at 24 h: *p* = 0.0009). Pielou’s evenness also declined under nutrient enrichment, with differences becoming significant after 72 h (C^+^: *p* = 0.0004; P^+^: *p* = 0.0103; Figure 1E).

### 3.2 Nutrient enrichment induces ectodermal mucus shedding and restructures surface microbiota

Despite substantial microbiome restructuring, exposure to elevated nutrient concentrations did not induce visible disease symptoms in *Nematostella* as has been described for the fresh water polyp *Hydra* under elevated nutrient conditions (Lachnit et al., 2025). However, we observed a previously undescribed behavior characterized by rolling of the ectodermal mucus layer along the body column, formation of a detachable ring, and subsequent shedding at the oral end (Supplementary Video 1). SYBR Gold staining combined with epifluorescence microscopy indicated a strong reduction of surface-associated bacterial signal on polyps immediately following shedding, while shed mucus layers exhibited dense bacterial staining (Supplementary Figure 1 and Supplementary Videos 2, 3).

**FIGURE 2:**
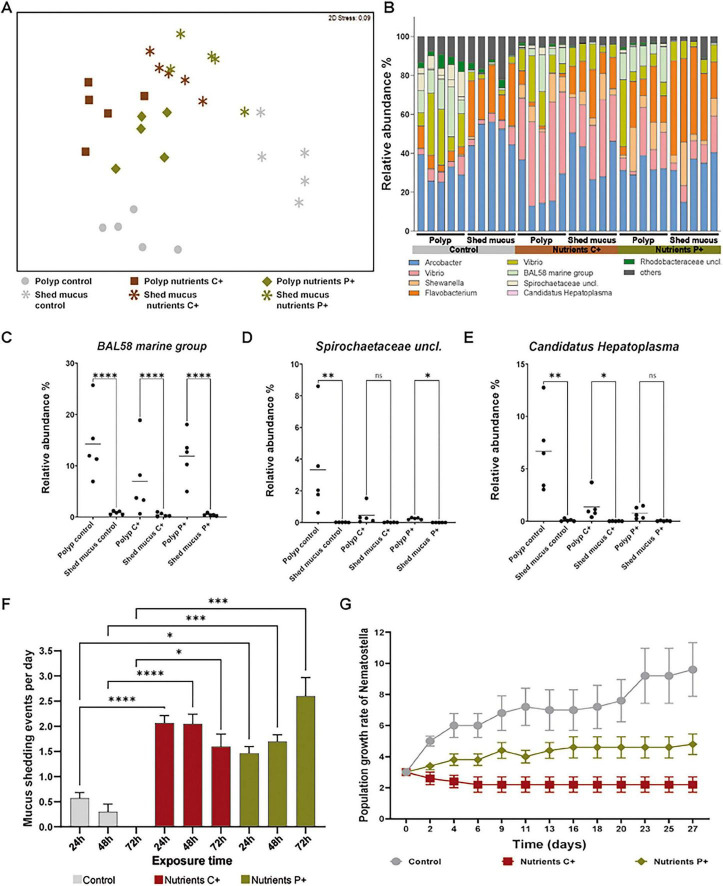
Nutrient enrichment induces ectodermal mucus shedding as microbial regulation with physiological impact on the host. **(A)** Non-metric multidimensional scaling analysis (nMDS) of bacterial communities associated with polyps and shed mucus under control, complex nutrient (C^+^), and protein-rich nutrient (P^+^) conditions. Mucus-associated communities were clearly distinct from polyp-associated communities in all treatments (PERMANOVA pairwise comparisons: control *t* = 3.88, *p* = 0.007; C^+^ *t* = 2.68, *p* = 0.009; P^+^ *t* = 2.53, *p* = 0.011). **(B)** Relative abundance of dominant bacterial operational taxonomic units (OTUs) polyps and shed mucus under control, complex nutrient (C^+^), and protein-rich nutrient (P^+^) conditions. **(C–E)** Comparison of microbial communities between polyps and corresponding shed mucus. Certain taxa, including the *BAL58 marine group*, *Candidatus Hepatoplasma*, and *Spirochaetaceae*, were largely retained in polyp tissue but largely absent from shed mucus, indicating selective microbial retention. Data are shown as original values. Statistical analyses were performed on LN-transformed data using one-way ANOVA followed by Šidák multiple comparisons. *Spirochaetaceae* did not meet assumptions of normality or homogeneity of variances after transformation, non-parametric Kruskal–Wallis test followed by Dunn’s multiple comparison tests were applied. **(F)** Mucus shedding frequency over 72 h. Both nutrient-enriched treatments exhibited significantly higher shedding rates than controls at all time points (Kruskal–Wallis tests *p* < 0.0001; Dunn’s multiple comparisons test: C^+^ 24 and 48 h *p* < 0.0001, 72 h *p* = 0.049; P^+^ 24 h *p* = 0.0283, 48 h *p* = 0.0024, 72 h *p* = 0.0001). Frequency in controls declined over time, while nutrient-enriched animals maintained high rates. **(G)** Population growth over 27 days. Control populations increased in size, whereas protein-rich nutrient conditions reduced growth and complex nutrient conditions resulted in net population decline, demonstrating the physiological costs associated with repeated mucus shedding under nutrient enrichment. Values represent mean ± SE. The symbols *, **, ***, and **** denote statistical significance at *p* < 0.05, *p* < 0.01, *p* < 0.001, and *p* < 0.0001, respectively.

This qualitative observation suggests redistribution of bacteria during shedding; however, absolute bacterial abundance was not quantified. Selective collection of shed mucus rings and polyps followed by 16S rRNA gene sequencing revealed that bacterial community composition differed significantly between mucus associated and polyps within the same environment. Non-metric multidimensional scaling analyses (nMDS) illustrated clear separation of shed mucus rings and polyp-associated communities (Figure 2A), which was confirmed by PERMANOVA pairwise-test (control: *t* = 3.8774, *p* = 0.007; C^+^: *t* = 2.6849, *p* = 0.009; P^+^: *t* = 2.5318, *p* = 0.011). Shed mucus from control polyps were dominated by five major OTUs, including *Arcobacter*, two *Vibrio* OTUs, *Flavobacterium*, and members of the *Rhodobacteraceae*. Under nutrient-enriched conditions, the bacterial composition of shed mucus rings shifted, with increased relative abundance of *Shewanella* and a concomitant decrease in *Rhodobacteraceae* compared to control mucus samples (Figure 2B).

Comparison of bacterial communities from shed mucus rings and whole polyps revealed several OTUs that were closely associated with polyp tissue but were largely absent from mucus. These included the *BAL58 marine group* (< 2%), *Candidatus Hepatoplasma*, and members of the *Spirochaetaceae* (both ∼0%) (Figures 2C–E).

### 3.3 Nutrient enrichment increases mucus shedding frequency and reduces population growth

Quantification of mucus shedding over 72 h revealed that, although occasional mucus shedding occurred in control animals, shedding frequency was markedly higher under both nutrient-enriched conditions, exceeding two sheddings per polyp per day (Figure 2F). Non-parametric Kruskal–Wallis followed by Dunn’s multiple comparisons test showed that mucus shedding frequency was significantly higher than controls at all time points in both C^+^ (24 and 48 h: *p* < 0.0001; 72 h: *p* = 0.049) and P^+^ (24 h: *p* = 0.0283; 48 h: *p* = 0.0024 and 72 h: *p* = 0.0001) treatments, whereas no significant difference was observed between C^+^ and P^+^ (*p* > 0.054). Mucus shedding frequency in controls declined over time, approaching zero by 72 h. In contrast, frequency increased over time in the P^+^ treatment. C^+^ animals exhibited higher initial shedding rates than P^+^, followed by a modest decline between 48 and 72 h (Figure 2F).

To assess potential physiological costs associated with increased shedding, we conducted a population growth experiment over 27 days (Figure 2G). Groups of three *Nematostella* individuals were maintained under control, C^+^, or P^+^ conditions (*n* = 5 replicates per treatment) and fed twice per week. Population growth differed significantly among treatments over time [mixed-effects model, treatment × time interaction: *F*(24, 143) = 6.001, *p* < 0.0001]. Both time [*F*(1.510, 18.00) = 8.493, *p* = 0.0045] and treatment [*F*(2, 12) = 10.42, *p* = 0.0024] also had significant effects on population size. Control populations exhibited the highest growth over the 27–day period, reaching an average of 9.6 individuals per container. In contrast, populations exposed to protein-rich medium showed reduced growth, while complex nutrient enrichment resulted in the strongest suppression of population expansion. Šidák’s multiple comparisons test revealed that control populations were significantly larger than C^+^ populations at multiple time points throughout the experiment, with differences becoming more pronounced over time. P^+^ treatments showed intermediate effects, with significant differences from controls occurring at fewer time points, while direct comparisons between C^+^ and P^+^ were only occasionally significant at later stages of the experiment.

## 4 Discussion

**FIGURE 3:**
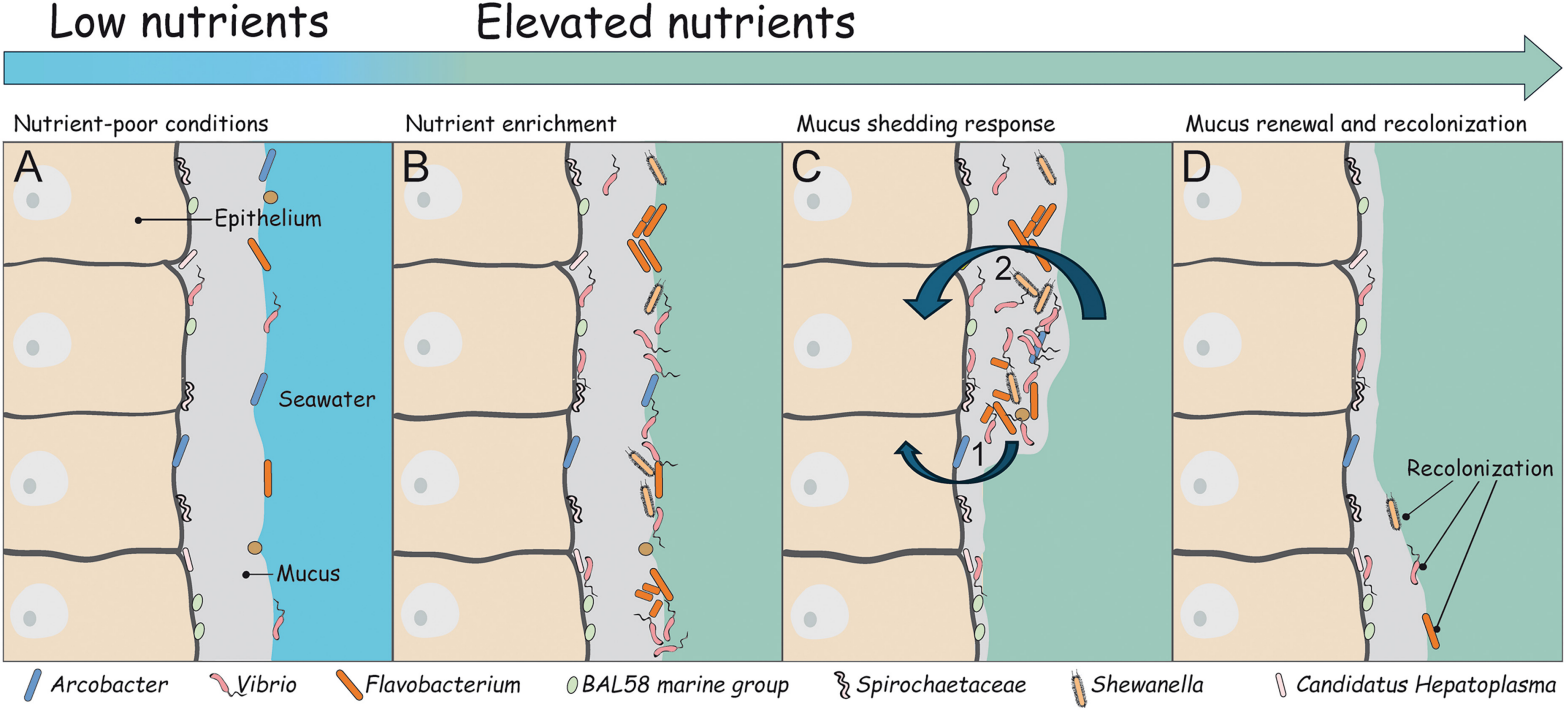
Graphical illustration and interpretation of the experimental results. **(A)** Baseline condition (clean seawater). *Nematostella* polyps maintained in clean, nutrient-free artificial seawater harbor a microbiota that is spatially structured within the mucus layer. Differences between microbiome sequencing of the mucus and whole polyps indicate that some bacteria are located close to the epithelial surface while others inhabit the outer mucus layer. Under these oligotrophic conditions, host-secreted metabolites are likely the primary nutrient source for associated microbes. This suggests that the host has strong potential to regulate bacterial abundance and community composition through nutrient availability and the composition of released metabolites. **(B)** Nutrient enrichment. Elevated nutrient concentrations in the surrounding seawater lead to shifts in bacterial community composition, characterized by an increase in copiotrophic bacteria. The higher abundance of these taxa under nutrient-rich conditions suggests that their low abundance under clean conditions is not primarily controlled by host antimicrobial mechanisms (e.g., antimicrobial peptides), but rather by nutrient limitation. External nutrient supply appears to relieve this limitation and promotes bacterial growth. **(C)** Mucus shedding. Under elevated nutrient conditions, the mucus layer is periodically shed and removed at the oral pole of the polyp, together with its associated microbial community. At present, it can only be hypothesized that increased bacterial abundance and their metabolic by-products trigger this mucus shedding as a host defense response (C1), or that elevated nutrient levels directly stimulate mucus production and shedding (C2). **(D)** Mucus recovery and recolonization. Following shedding, the mucus layer is regenerated and recolonized by bacteria. If nutrient concentrations remain elevated, bacterial populations rapidly increase again, leading to renewed overgrowth and the initiation of another mucus shedding event.

Nutrient enrichment in the environment dramatically restructured the microbiome of *Nematostella vectensis*. This alteration was accompanied by a marked increase in ectodermal mucus shedding and reduced population growth. Together, these findings demonstrate that elevated dissolved nutrients are sufficient to alter host-associated microbial community composition and induce a pronounced epithelial response. Conceptually, our results support a model in which nutrient availability structures host–microbe interactions at the epithelial surface (Figure 3). Under nutrient-poor conditions, microbial growth is likely constrained by host-derived metabolites released into the mucus layer, providing the host with indirect control over its microbiota. External nutrient enrichment relaxes this limitation, allowing fast-growing copiotrophic bacteria to proliferate and restructure the microbiome. While the mechanistic links among nutrient availability, microbiome restructuring, and host fitness cannot be fully disentangled in the present study, the data support a model in which nutrient enrichment destabilizes host–microbiome homeostasis and elicits compensatory host responses that carry physiological costs, potentially linking nutrient enrichment to eutrophication driven host–microbe imbalance. Importantly, we interpret our findings primarily as microbial restructuring under nutrient enrichment. Although the observed reduction in diversity and dominance of copiotrophic taxa are consistent with commonly used operational definitions of dysbiosis, overt disease symptoms were not observed. Thus, nutrient enrichment induced a state of altered microbiome structure and reduced diversity rather than clear pathological dysbiosis.

### 4.1 Nutrient availability and composition restructure the *Nematostella* microbiome

Exposure to elevated dissolved nutrient concentrations induced pronounced and reproducible shifts in bacterial community composition, with both enriched treatments diverging strongly from controls and from each other. These findings indicate that not only nutrient load but also nutrient quality structure host-associated microbial assemblages, consistent with patterns reported for other invertebrates under eutrophication or nutrient enrichment (Wang et al., 2018; Klinges et al., 2023; Lachnit et al., 2025).

Prior studies have shown that *Nematostella* maintains relatively stable core microbial communities under controlled laboratory conditions while retaining environmental responsiveness (Mortzfeld et al., 2016; Baldassarre et al., 2022; Domin et al., 2023). Our results are consistent with this framework: nutrient enrichment did not randomize the microbiome but shifted relative abundances in a structured manner, favoring specific bacterial groups. In particular, enrichment resulted in a marked dominance of copiotrophic taxa such as *Vibrio*, *Shewanella* and *Flavobacterium*, bacteria that are commonly associated with nutrient-rich environments and rapid exploitation of dissolved organic matter (Dai et al., 2022). The increased abundance of these taxa under nutrient-enriched conditions suggests that their typically low representation under control conditions is not primarily constrained by host antibacterial defense mechanisms, such as pattern-recognition receptor signaling or the production of antibacterial effector molecules (Parisi et al., 2020), but more likely by limited nutrient availability within the host-associated microenvironment. Consistent with this idea, recolonization studies in *Nematostella vectensis* have demonstrated that early microbial colonization is strongly influenced by host-derived substrates, whereas subsequent community succession is largely driven by bacteria-bacteria interactions (Domin et al., 2023). External nutrient supply may therefore relieve this limitation and promote rapid bacterial proliferation. This interpretation supports the view that resource availability, rather than direct host suppression alone, can play a central role in structuring host-associated microbial communities.

At the same time, we observed a decrease in the relative abundance of taxa such as *Spirochaetota*, *BAL58 marine group* and *Candidatus Hepatoplasma*. Importantly, reductions in relative abundance do not necessarily indicate absolute declines; rather, these taxa may have maintained stable population sizes while being outcompeted numerically by fast-growing copiotrophs. Nevertheless, their reduced proportional representation suggests limited capacity to exploit external nutrient pulses. This pattern is consistent with the hypothesis that many members of the native *Nematostella* microbiome are adapted to nutrient-limited conditions within the host mucus environment. In such a scenario, host-secreted metabolites may represent the dominant nutrient source, effectively coupling microbial growth to host physiology. External nutrient enrichment would therefore decouple this relationship by providing readily accessible substrates from the surrounding environment, enabling opportunistic bacteria to outcompete host-adapted taxa.

Some members of the native microbiome may therefore rely more strongly on host-derived resources such as mucus associated polysaccharides, proteins or lipids as described for other cnidarians (Krediet et al., 2009; Hubot et al., 2026). This nutrient dependency may be particularly relevant for *Spirochaetes*, which have been shown to associate with adult polyps and oocytes of *Nematostella*, suggesting potential vertical transmission (Baldassarre et al., 2021). The shift toward fast-growing taxa under nutrient enrichment mirrors general ecological rules described for global marine bacterial communities, where nutrient-rich environments favor bacteria with rapid growth strategies, while nutrient-poor environments select for efficient, slower-growing organisms (Dai et al., 2022). The concomitant reductions in richness, diversity, and evenness observed here further support the interpretation that nutrient enrichment promotes community simplification and dominance by a small number of competitive taxa, a hallmark of dysbiosis across diverse host–microbiome systems (Patterson et al., 2015; Egan and Gardiner, 2016).

### 4.2 Mucus shedding as a host-mediated mechanism of microbial regulation

Despite extensive microbiome restructuring, polyps exposed to nutrient enriched environments did not display overt disease symptoms, as previously reported for the freshwater polyp *Hydra* under nutrient rich conditions (Lachnit et al., 2025). Instead, they exhibited a striking ectodermal mucus shedding behavior that led to physical removal of surface-associated bacteria, with shed mucus layers carrying the majority of external microbes. Although qualitative, this response suggests that shedding serves as a rapid mechanism to regulate microbial load when external conditions favor excessive bacterial proliferation.

The present study cannot distinguish whether mucus shedding is directly triggered by elevated nutrient concentrations or indirectly induced by the proliferation and metabolic activity of mucus-associated bacteria, because the germ-free or controlled recolonization experiments required to disentangle these mechanisms were beyond the scope of the present study. Two non-mutually exclusive mechanisms may explain this response (Figure 3C). First, increased bacterial abundance and associated metabolic activity may trigger epithelial shedding as a defensive response to microbial overgrowth. Alternatively, elevated nutrient concentrations may directly stimulate mucus production and shedding independent of microbial signals. Resolving these possibilities will require experiments using germ-free animals or controlled microbial colonization. Although active mucus shedding has not, to our knowledge, been previously described for *Nematostella*, comparable strategies of microbial regulation have been reported in other cnidarians and marine invertebrates (Sweet et al., 2011; Glasl et al., 2016, 2017; Marchioro et al., 2020). In reef-building corals, aged mucus layers characterized by elevated prokaryotic abundance and increased prevalence of opportunistic or potentially pathogenic bacteria are regularly shed, contributing to pathogen defense and microbiome restructuring (Glasl et al., 2016). These parallels suggest that mucus shedding may present a conserved mechanism of microbial regulation among cnidarians.

Distinct bacterial communities associated with shed mucus rings versus whole polyps further support the hypothesis of selective microbial retention at the host surface. Several taxa, including *Spirochaetaceae* and *Candidatus Hepatoplasma*, were largely absent from shed mucus but remained associated with polyp tissue, implying occupation of protected niches such as deeper mucus strata or epithelial-associated microhabitats, analogous to spatially structured symbionts described in corals (Apprill et al., 2016; Marcelino et al., 2018). Earlier work has shown that the *Nematostella* microbiome varies along the body column, indicating axial spatial organization of host-associated microbes. Our results suggest an additional layer of spatial structure across the mucus layer itself (Bonacolta et al., 2021). Taxa such as *Spirochaetaceae* and *Candidatus Hepatoplasma* remained associated with polyps but were largely absent from shed mucus, consistent with localization closer to the epithelial surface. In contrast, taxa enriched under nutrient conditions, particularly *Shewanella* and *Vibrio* were disproportionately represented in shed mucus, suggesting a more loosely attached surface-associated lifestyles with the outer mucus layer. Together, these observations indicate that microbial communities are structured both along the body axis and across the mucus layer. Mucus shedding therefore likely represents more than passive sloughing of surface material; rather, it appears to be a plastic and environmentally responsive host defense that links external nutrient conditions to regulation of microbial load. However, whether this process preferentially removes opportunistic bacteria proliferating under nutrient-rich conditions or instead reflects a broader physiological response to nutrient exposure remains unresolved.

### 4.3 Ecological and physiological costs of increased mucus shedding

While mucus shedding appears to protect against microbial overgrowth, the population growth experiments indicate that this response is accompanied by substantial costs. Both nutrient-enriched treatments displayed reduced population growth relative to controls, with the strongest negative effect observed under complex nutrient enrichment. Increased shedding frequency likely imposes energetic costs associated with mucus production, epithelial regeneration and the repeated loss and re-assembly of beneficial microbial partners, similar to the energetic and physiological burdens associated with repeated mucus release in corals under stress (Wooldridge, 2009; Savoca et al., 2022).

The divergent population trajectories between complex and protein-rich media treatments further suggest that the balance between nutritional benefits and physiological costs depends on nutrient composition: while protein-rich conditions supported some population growth, complex enrichment resulted in net decline, potentially reflecting stronger microbial proliferation, more intense shedding responses, or more pronounced disruption of beneficial microbial functions.

### 4.4 Implications for host–microbiome stability under nutrient enrichment

Overall, these findings indicate that nutrient enrichment restructures the *Nematostella* microbiome by promoting opportunistic, copiotrophic bacteria and reducing diversity, thereby triggering host-mediated responses that mitigate microbial overgrowth but at significant physiological cost. In natural estuarine and coastal habitats, ongoing eutrophication may thus place cnidarians under chronic microbial stress, forcing repeated cycles of microbial proliferation, mucus shedding and microbiome reassembly (Figure 3D). Over time, such cycles can erode host fitness even in the absence of overt disease (Bourne et al., 2016; Zaneveld et al., 2016; Vega Thurber et al., 2020).

By experimental linking nutrient enrichment to microbiome restructuring, epithelial mucus shedding, and reduced clonal growth in a tractable model cnidarian, this study provides mechanistic insights into how eutrophication can influence host–microbe interactions. In this context, shedding emerges as a key, yet costly, mechanism by which *Nematostella* dynamically regulate its microbiota in a changing nutritional landscape, complementing immune and epithelial processes described in other cnidarian models such as *Hydra* and reef-building corals (Fraune and Bosch, 2007; Brown and Bythell, 2005; Bosch and McFall-Ngai, 2011).

Future experiments integrating quantitative microbial load measurements, germ-free manipulations, and host transcriptomics will be essential to resolve causal pathways and determine whether mucus shedding represents primarily a nutrient stress response, a microbiome-regulatory mechanism, or an integrated response to both factors.

## Data Availability

The datasets presented in this study can be found in online repositories. The names of the repository/repositories and accession number(s) can be found below: https://www.ncbi.nlm.nih.gov/, SRX31876269–SRX31876193.
