# Supplementary material for: Nutrient-rich environments drive microbiome restructuring and mucus shedding in a coastal cnidarian
Source: Front Microbiol. 2026 May 18;17:1792133. doi: 10.3389/fmicb.2026.1792133 (PMC13223050; doi:10.3389/fmicb.2026.1792133)
Supplement: Supplementary file 1 [file Table_1.docx]

| **Control & Complex medium (C⁺)** |  |  |  |  |  |  |
| --- | --- | --- | --- | --- | --- | --- |
| Average dissimilarity = 46.36 |  |  |  |  |  |  |
|  | **Control** | **Complex medium (C⁺)** |  |  |  |  |
| **Species** | **Av. Abund** | **Av. Abund.** | **Av.Diss** | **Diss/SD** | **Contrib%** | **Cum.%** |
| Vibrio Otu5 | 6.08 | 21.56 | 7.74 | 6.45 | 16.7 | 16.7 |
| Shewanella Otu7 | 0.75 | 12.6 | 5.92 | 4.21 | 12.78 | 29.47 |
| BAL58_marine_group Otu18 | 11.35 | 6.06 | 3.11 | 1.62 | 6.71 | 36.18 |
| Spirochaetaceae Otu35 | 7.34 | 1.33 | 3.04 | 1.85 | 6.56 | 42.73 |
| Flavobacterium Otu9 | 4.14 | 8.2 | 2.67 | 1.31 | 5.75 | 48.49 |
| Candidatus_Hepatoplasma Otu50 | 6.67 | 2.19 | 2.34 | 2.12 | 5.05 | 53.54 |
| Rhodobacteraceae_unclassified Otu51 | 5.88 | 1.81 | 2.08 | 2.02 | 4.48 | 58.02 |
| Francisella Otu65 | 4.93 | 1.02 | 2.02 | 1.76 | 4.36 | 62.38 |
| Vibrio Otu14 | 10.67 | 13.38 | 1.81 | 1.51 | 3.91 | 66.3 |
| Arcobacter Otu3 | 13.86 | 15.47 | 1.56 | 1.34 | 3.36 | 69.66 |
| Pseudoalteromonas Otu87 | 0.93 | 2.41 | 1.08 | 1.08 | 2.32 | 71.98 |
|  |  |  |  |  |  |  |
| **Control & Protein-rich medium (P⁺)** |  |  |  |  |  |  |
| Average dissimilarity = 48.49 |  |  |  |  |  |  |
|  | **Control** | **Protein-rich medium (P⁺)** |  |  |  |  |
| **Species** | **Av. Abund** | **Av. Abund** | **Av.Diss** | **Diss/SD** | **Contrib%** | **Cum.%** |
| Shewanella Otu7 | 0.75 | 18.02 | 8.63 | 2.81 | 17.8 | 17.8 |
| Flavobacterium Otu9 | 4.14 | 16.73 | 6.3 | 2.47 | 12.99 | 30.8 |
| Spirochaetaceae Otu35 | 7.34 | 0.73 | 3.3 | 2.13 | 6.82 | 37.61 |
| Vibrio Otu5 | 6.08 | 10.95 | 2.66 | 1.53 | 5.49 | 43.11 |
| Candidatus_Hepatoplasma Otu50 | 6.67 | 1.65 | 2.51 | 2.48 | 5.18 | 48.29 |
| BAL58_marine_group Otu18 | 11.35 | 8.23 | 2.5 | 1.22 | 5.16 | 53.45 |
| Rhodobacteraceae_unclassified Otu51 | 5.88 | 1.47 | 2.24 | 2.03 | 4.62 | 58.07 |
| Arcobacter Otu3 | 13.86 | 17.15 | 2.02 | 1.87 | 4.18 | 62.25 |
| Francisella Otu65 | 4.93 | 1.42 | 1.92 | 1.67 | 3.95 | 66.2 |
| Vibrio Otu33 | 5.48 | 2.52 | 1.63 | 1.57 | 3.36 | 69.56 |
| Vibrio Otu14 | 10.67 | 12.28 | 1.6 | 1.37 | 3.3 | 72.86 |
|  |  |  |  |  |  |  |
| **Complex medium (C⁺) & Protein-rich medium (P⁺)** |  |  |  |  |  |  |
| Average dissimilarity = 32.04 |  |  |  |  |  |  |
|  | **Complex medium (C⁺)** | **Protein-rich medium (P⁺)** |  |  |  |  |
| **Species** | **Av. Abund.** | **Av. Abund** | **Av.Diss** | **Diss/SD** | **Contrib%** | **Cum.%** |
| Vibrio Otu5 | 21.56 | 10.95 | 5.3 | 2.56 | 16.55 | 16.55 |
| Flavobacterium Otu9 | 8.2 | 16.73 | 4.49 | 1.63 | 14.01 | 30.56 |
| Shewanella Otu7 | 12.6 | 18.02 | 3.72 | 1.69 | 11.6 | 42.16 |
| BAL58_marine_group Otu18 | 6.06 | 8.23 | 2.91 | 1.49 | 9.09 | 51.25 |
| Vibrio Otu33 | 7.27 | 2.52 | 2.38 | 2.01 | 7.44 | 58.68 |
| Arcobacter Otu3 | 15.47 | 17.15 | 1.85 | 1.5 | 5.79 | 64.47 |
| Pseudoalteromonas Otu87 | 2.41 | 1.99 | 1.45 | 0.99 | 4.51 | 68.98 |
| Vibrio Otu14 | 13.38 | 12.28 | 1.36 | 1.43 | 4.24 | 73.22 |

**Supplementary Table S1**: SIMPER analysis pairwise tests of differences between clean water controls and different nutrient treatments Complex medium (C⁺) & Protein-rich medium (P⁺), based on averages across all replicates and time points.
