# Supplementary material for: Nutrient-rich environments drive microbiome restructuring and mucus shedding in a coastal cnidarian
Source: Front Microbiol. 2026 May 18;17:1792133. doi: 10.3389/fmicb.2026.1792133 (PMC13223050; doi:10.3389/fmicb.2026.1792133)
Supplement: Supplementary file 5 [file Data_Sheet_1.docx]

**Supplementary Figure S1:** SYBR Gold–stained surface-associated bacteria of *Nematostella vectensis* after 24 h exposure to different nutrient regimes in the surrounding water. Images on the left were obtained using a combination of epifluorescence and light microscopy, whereas images on the right were acquired using epifluorescence microscopy only to enhance the visibility of bacterial cells. (A, B) Control polyps exposed to clean seawater, showing only a few bacterial cells attached to the surface epithelial layer and no molt formation. (C–H) Polyps exposed to elevated nutrient conditions. Under nutrient-enriched conditions, surface epithelial shedding was observed, characterized by the formation of ring-like structures filled with SYBR Gold-stained bacteria (green). Shedded material surrounding the polyps was visible. Surface areas of the freshly shed epithelial areas from nutrient-exposed polyps showed reduced bacterial coverage.

**
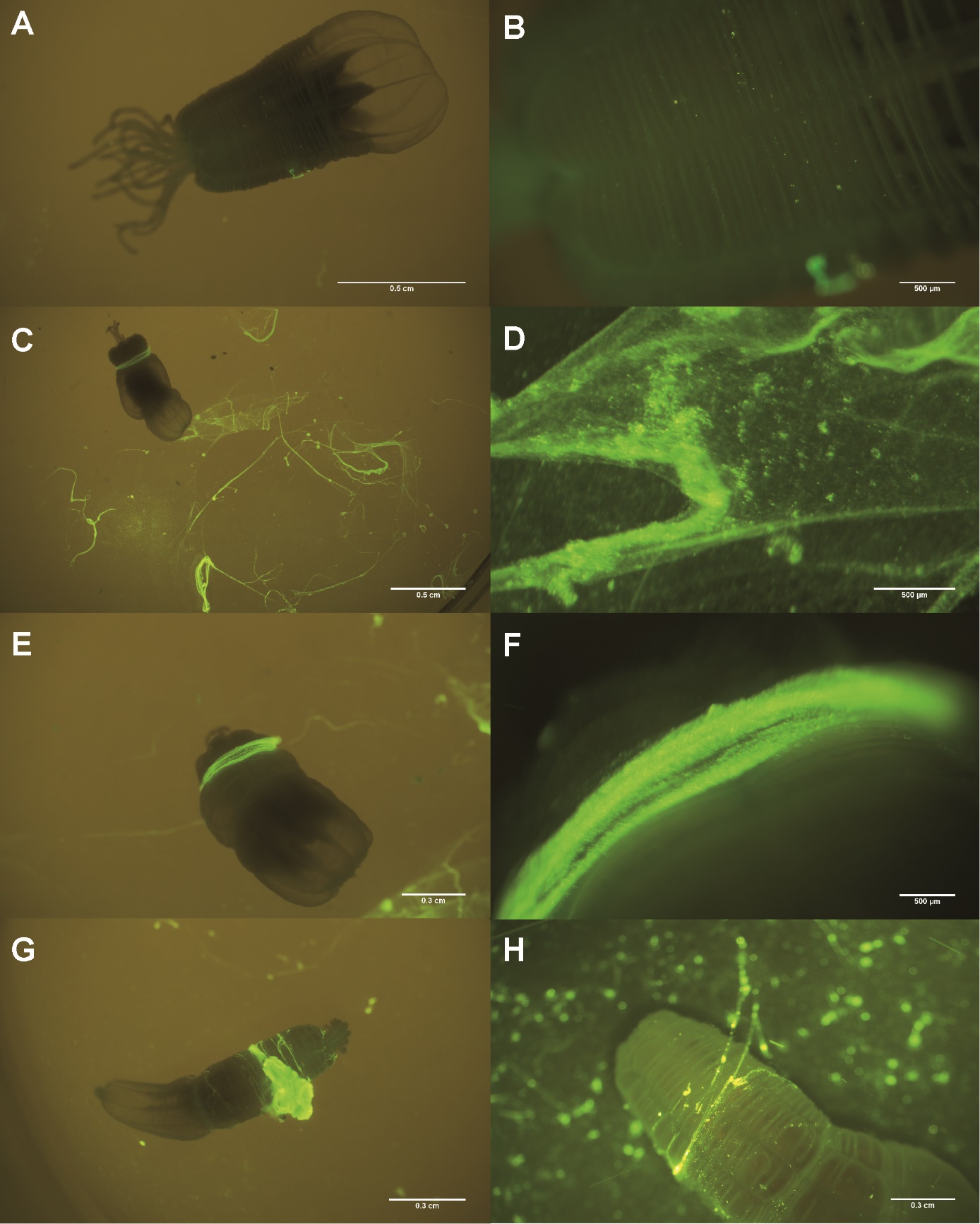
**
